# G protein-coupled estrogen receptor activates PI3K/AKT/mTOR signaling to suppress ferroptosis via SREBP1/SCD1-mediated lipogenesis

**DOI:** 10.1186/s10020-023-00763-x

**Published:** 2024-02-21

**Authors:** Jiaping Chen, Rong Zhao, Yangwei Wang, Han Xiao, Wei Lin, Mingxin Diao, Shiwen He, Peiyuan Mei, Yongde Liao

**Affiliations:** 1 Department of Thoracic Surgery, Union Hospital, Tongji Medical College, Huazhong University of Science and Technology, Wuhan, 430022 China; 2 Department of Cardiothoracic Surgery, Third Affiliated Hospital of Kunming Medical University (Yunnan Cancer Hospital), Kunming, China; 3 Department of Thoracic Surgery, Shandong Provincial Hospital Affiliated to Shandong First Medical University, Jinan, China

**Keywords:** NSCLC, GPER1, Ferroptosis, Cisplatin and SCD1

## Abstract

**Background:**

Lung cancer is the leading cause of cancer-related death worldwide. The sex differences in the occurrence and fatality rates of non-small cell lung cancer (NSCLC), along with its association with estrogen dependence, suggest that estrogen receptors (ERs) contribute to the development of NSCLC. However, the influence of G protein-coupled estrogen receptor (GPER1) on NSCLC remains to be determined. Escape from ferroptosis is one of the hallmarks of tumor discovered in recent years. In this context, the present study evaluated whether GPER1 promotes NSCLC progression by preventing ferroptosis, and the underlying mechanism through which GPER1 protects against ferroptosis was also explored.

**Methods:**

The effects of GPER1 on the cytotoxicity of H_2_O_2_, the ferroptosis inducer RSL3, and Erastin were assessed using the CCK8 assay and plate cloning. Lipid peroxidation levels were measured based on the levels of MDA and BODIPY™581/591C11. GPER1 overexpression and knockdown were performed and G1 was used, and the expression of SCD1 and PI3K/AKT/mTOR signaling factors was measured. Immunofluorescence analysis and immunohistochemistry were performed on paired specimens to measure the correlation between the expression of GPER1 and SCD1 in NSCLC tissues. The effect of GPER1 on the cytotoxicity of cisplatin was measured in vitro using the CCK8 assay and in vivo using xenograft tumor models.

**Results:**

GPER1 and G1 alleviated the cytotoxicity of H_2_O_2_, reduced sensitivity to RSL3, and impaired lipid peroxidation in NSCLC tissues. In addition, GPER1 and G1 promoted the protein and mRNA expression of SCD1 and the activation of PI3K/AKT/mTOR signaling. GPER1 and SCD1 expression were elevated and positively correlated in NSCLC tissues, and high GPER1 expression predicted a poor prognosis. GPER1 knockdown enhanced the antitumor activity of cisplatin in vitro and in vivo.

**Conclusion:**

GPER1 prevents ferroptosis in NSCLC by promoting the activation of PI3K/AKT/mTOR signaling, thereby inducing SCD1 expression. Therefore, treatments targeting GPER1 combined with cisplatin would exhibit better antitumor effects.

**Graphical Abstract:**

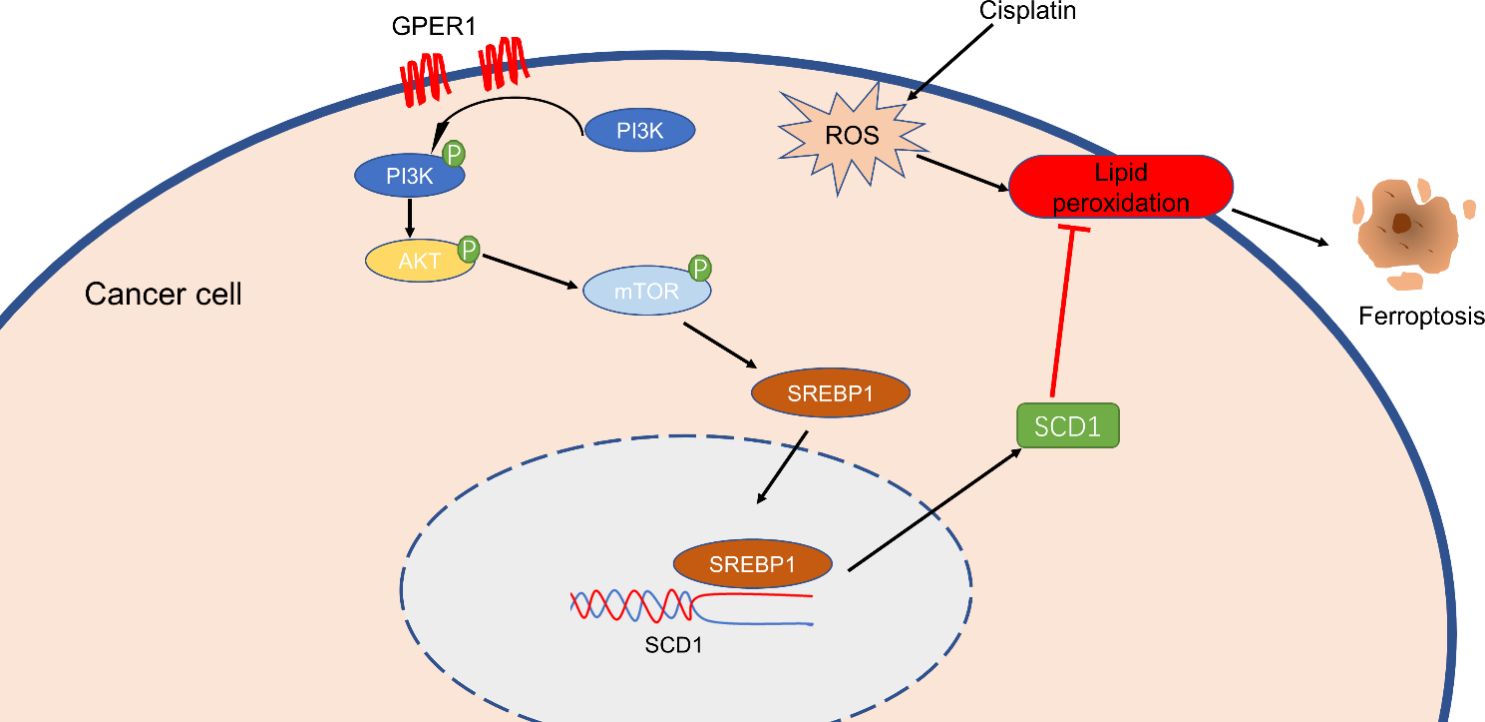

**Supplementary Information:**

The online version contains supplementary material available at 10.1186/s10020-023-00763-x.

## Introduction

Lung cancer is the primary cause of cancer-related deaths worldwide in men and women (Sung et al. 2021). Non-small cell lung cancer (NSCLC) accounts for 85% of all cases of lung cancer (Zhang et al., 2021). Researchers have been tirelessly searching for novel therapeutic targets to combat NSCLC. Recently, research has presented compelling evidence linking estrogen to NSCLC. A study conducted on 6500 breast cancer survivors revealed that women who received antiestrogen therapy exhibited a lower mortality rate due to lung cancer (Bouchardy et al. 2011). A study conducted with over 16,000 postmenopausal women who were treated with a placebo or daily hormone replacement therapy (HRT) for 5 years revealed that those in the HRT group exhibited a higher incidence of lung cancer morbidity and mortality (Chlebowski et al. 2009). Furthermore, prospective investigations have demonstrated that the risk of HRT increasing the incidence and mortality of lung cancer was time-dependent (Slatore et al. 2010). These findings suggest that estrogens play a role in tumor etiology and progression to a certain extent.

Estrogen is known to play a crucial role in regulating numerous physiological processes under normal conditions. Consequently, disruptions in the estrogen signaling pathway could lead to a variety of diseases, including reproductive system-related conditions such as endometriosis, bone-related abnormalities such as osteoporosis, and tumor-related conditions such as lung and breast cancer. In addition, estrogen abnormalities may contribute to the development of diseases related to the cardiovascular and digestive systems (Chen et al. 2022; Iorga et al. 2017). Estrogens function by binding to their receptors and activating them. These receptors include the classical ERα and ERβ, as well as the membrane-bound G protein-coupled estrogen receptor (GPER1) (Barton et al. 2018; Luo and Liu, 2020). Over the past two decades, extensive research has been conducted on ERα, ERβ, and estrogen signaling in relation to lung adenocarcinoma, which has a higher incidence in women (Garon et al. 2013; Marquez-Garban et al. 2007; Toh et al. 2010). However, research focused on GPER1 in lung cancer has been scarce.

The GPER1 protein is known to be biologically active and highly expressed in a range of solid tumors, particularly those with sex-based differences in their incidence rates, such as breast, endometrial, thyroid, and colon cancer (Hernandez-Silva et al. 2020; Hsu et al. 2019; Jacenik et al. 2019; Xu et al. 2019). Recently, research revealed that GPER1 expression was elevated in NSCLC compared to normal lung tissue (Jala et al. 2012), and higher expression correlated with poor postoperative prognosis in NSCLC patients (Li et al. 2022). Studies have indicated that the activation of EGFR via GPER1 leads to the phosphorylation of ERK1/2, which promotes the expression of matrix metalloproteinase-2 (MMP-2) and matrix metalloproteinase-9 (MMP-9). This, in turn, promotes NSCLC invasion and metastasis (Zhang et al. 2014). Recent studies have demonstrated that GPER1 interacts with E2 or fulvestrant, which are antagonists of ERα and ERβ, respectively, to activate and promote LUAD cell proliferation in vitro and in vivo (Liu et al. 2015, 2019; Shen et al. 2021). Further research is necessary to elucidate the mechanism through which GPER1 promotes NSCLC progression. In this context, the present study aimed to investigate the specific mechanism through which GPER1 contributes to NSCLC progression.

Ferroptosis is a type of nonapoptotic cell death that relies on iron and may be detected based on lipid peroxidation (Dixon et al. 2012). Recent studies have suggested that inducing ferroptosis in tumor cells is an effective strategy to terminate tumor growth. In addition, inhibiting lipid peroxidation or reducing ferroptosis through pharmaceutical or genetic means could effectively prevent ferroptosis (Jiang et al. 2021; Zhang et al. 2022). Stearoyl CoA desaturase 1 (SCD1) is a crucial enzyme that catalyzes the conversion of saturated fatty acids (SFAs) into monounsaturated fatty acids (MUFAs). SCD1 is highly expressed in various tumors and reportedly promotes tumor progression. Furthermore, recent research revealed that SCD1 protects against tumor ferroptosis (Chen et al. 2021; Magtanong et al. 2019). In the context of pancreatic cancer, FBW7 plays a crucial role in regulating lipid peroxidation and promoting ferroptosis by suppressing SCD1 expression. Mechanistically, this is accomplished by inhibiting nuclear receptor subfamily 4 group A member 1 (NR4A1). This suppression of SCD1 ultimately leads to the promotion of ferroptosis in pancreatic cancer cells (Ye et al. 2021). SCD1 knockdown in the NSCLC cell line A549 led to a noteworthy decrease in the cell growth rate, along with an increase in intracellular iron levels and lipid peroxidation. These findings indicate that SCD1 plays a crucial role in regulating ferroptosis (Jiang et al. 2017). Tumor cells with PI3K hyperactive mutations are highly resistant to ferroptosis. However, when the PI3K/AKT/mTOR signaling axis is inhibited, cancer cells become sensitive to ferroptosis. Mechanistic studies have demonstrated that this signaling axis protects cancer cells from ferroptosis by producing MUFAs via SREBP1/SCD1 (Yi et al. 2020). Previous investigations by our research group have revealed that estrogen exerts antioxidant effects on tumors while promoting the expression of SCD1 (Belkaid et al. 2015; Chen et al. 2013; Kong et al. 2014). This finding led to the hypothesis that estrogen could regulate tumor ferroptosis. To date, no study has investigated the potential association between GPER1 and SCD1. In the present study, it was hypothesized that GPER1 is linked to ferroptosis, and SCD1 plays a role in this process.

## Materials and methods

### Patients and samples

The set of samples used in the present study were 96 formalin-fixed, paraffin-embedded tissue samples from patients with primary NSCLC, who had been diagnosed at the Department of Thoracic Surgery of Union Hospital, Tongji Medical College, Huazhong University of Science and Technology (Wuhan, China), between the years 2014 and 2018. None of these patients had used hormone therapy, radiation, or chemotherapy prior to the surgery. Patients’ clinical details were noted, and at least two pathologists verified the diagnosis. At the Union Hospital, the pTNM stage and tumor differentiation grade were also noted. The respective tissue samples were obtained with informed consent from all patients. The baseline characteristics of the study participants are presented in Table 1.

**Table 1:** Clinical characteristics of patients

	Adenocarcinoma	Squamous	All
**All**	52(54.2%)	44(45.8%)	96(100%)
**Gender**			
Male	33(63.5%)	40(90.9%)	73(76.0%)
Female	19(36.5%)	4(9.1%)	23(24.0%)
**Age(years)**			
≤ 60	34(65.4%)	28(63.6%)	62(64.6%)
>60	18(34.6%)	16(36.4%)	34(35.4%)
**Tumor differentiation grade**			
Poor	12(23.2%)	15(34.1%)	27(28.1%)
Poor ~ moderate	19(36.5%)	12(27.3%)	31(32.3%)
Moderate	18(34.6%)	11(25%)	29(30.2%)
Well /Moderate-well	3(57.7%)	6(13.6%)	9(9.4%)
**Pathological tumor (T) status**			
T1 ~ T2	37(71.1%)	28(63.6%)	65(67.7%)
T3 ~ T4	15(28.9%)	16(36.4%)	31(32.3%)
**Pathological node (N) status**			
N0 ~ N1	15(28.9%)	23(52.3%)	38(39.6%)
N2 ~ N3	37(71.1%)	21(47.7%)	58(61.4%)
**Clinical stage**			
I ~ II	9(17.3%)	18(40.9%)	27(28.1%)
III	43(82.7%)	26(59.1%)	69(71.9%)

### Reagents and antibodies

G-1 (HY-107,216), RSL3 (HY-100,218 A), Erastin (HY-15,763), A939572 (HY-50,709), NSC781406 (HY-100,470), and Fatostatin (HY-14,452) were purchased from Med-Chem-Express. All these reagents were dissolved in DMSO, which was obtained from Servicebio (GC203005). The primary antibodies used in the present study included GPER1 (ABclonal, A10217), SCD1 (ABclonal, A16429), SREBP1 (Servicebio, GB113804), PI3K (ZENBIO, R22768), Phospho-PI3K (Tyr467/Tyr199) (ZENBIO, 310,164), Phospho-mTOR (Ser2448) (ZENBIO, 381,557), AKT (ABmart, T55561), Phospho-AKT (Ser473) (ABmart, T40067), GAPDH (ABclonal, AC033), mTOR (ABmart, T55306), GPX4 (ZENBIO, R381958), and NRF2 (ABmart, T55136). The second antibodies, horseradish peroxidase (HRP) goat anti-rabbit IgG (ABclonal, AS014) and (Servicebio, G1213), HRP goat anti-mouse IgG (ABclonal, AS003) were applied. A dilution ratio of 1:1000 was used in western blotting and 1:100 in immunohistochemistry and immunofluorescence analyses.

### Cell culture

The NSCLC cell lines A549 (CCL-185, ATCC) and H1299 (CRL-5803, ATCC) were obtained from ATCC. All cell lines were cultured in the RPMI-1640 medium (Gibico) supplemented with 10% fetal bovine serum (FBS; Gibico) and antibiotics (100 units/mL penicillin and 100 μg/mL streptomycin) using standard procedures. The cell culture was conducted in an incubator at 37 °C with 5% CO_2_ and 95% air atmosphere.

### Transfection

The shRNA lentiviral and GPER1 plasmid lentiviral with puromycin resistance vectors were obtained from Genechem (Shanghai, China). The cells were seeded in the wells of 6-well plates and then treated with puromycin for a minimum of 5 days after being infected with the lentiviral for 72 h. afterward, the transfection efficiency was verified through qRT-PCR and western blotting. The sequence for shGPER1 was 5′- CGG CTT TGT GGG CAA CAT C-3′, while 5′-TTC TCC GAA CGT GTC ACG T-3′ was used as the negative control.

### RNA extraction and qRT-PCR

Trelief^TM^-RNAprep-FastPure Tissue & Cell Kit (TSP413, TSINGKE) was employed for RNA extraction. The reverse transcription process was performed using the ABScript II cDNA First-Strand Synthesis Kit (RK20400, ABclonal). The subsequent qRT-PCR was conducted using the SYBR Green mix (G3326, Servicebio). The primers for the qPCR were obtained from TSINGKE, and the primer sequences are presented in Table 2.

**Table 2:** Primer sequences used for qRT-PCR

Name	Sequence (5’ ~ 3’)	Length
GPX4 (Forward Primer)	GAGGCAAGACCGAAGTAAACTAC	23
GPX4 (Reverse Primer)	CCGAACTGGTTACACGGGAA	20
NRF2 (Forward Primer)	TCAGCGACGGAAAGAGTATGA	21
NRF2 (Reverse Primer)	CCACTGGTTTCTGACTGGATGT	22
SLC7A11 (Forward Primer)	TCTCCAAAGGAGGTTACCTGC	21
SLC7A11 (Reverse Primer)	AGACTCCCCTCAGTAAAGTGAC	22
FTH1 (Forward Primer)	CCCCCATTTGTGTGACTTCAT	21
FTH1 (Reverse Primer)	GCCCGAGGCTTAGCTTTCATT	21
SCD1 (Forward Primer)	TCTAGCTCCTATACCACCACCA	22
SCD1 (Reverse Primer)	TCGTCTCCAACTTATCTCCTCC	22
CBS (Forward Primer)	GGCCAAGTGTGAGTTCTTCAA	21
CBS (Reverse Primer)	GGCTCGATAATCGTGTCCCC	20
FTMT (Forward Primer)	TGGAGTGTGCTCTACTCTTGG	21
FTMT (Reverse Primer)	ACGTGGTCACCTAGTTCTTTGA	22
MTOR (Forward Primer)	ATGCTTGGAACCGGACCTG	19
MTOR (Reverse Primer)	TCTTGACTCATCTCTCGGAGTT	22
GPER1 (Forward Primer)	CACCAGCAGTACGTGATCGG	20
GPER1 (Reverse Primer)	CATCTTCTCGCGGAAGCTGAT	21
GAPDH (Forward Primer)	GGAGCGAGATCCCTCCAAAAT	21
GAPDH (Reverse Primer)	GGCTGTTGTCATACTTCTCATGG	23

### Western blotting

The cells were fully lysed using the ice-cold RIPA lysis buffer [1% Nonidet P-40, 50 mM Tris-HCl (pH 7.4), 150 mM sodium chloride, and 0.5% sodium deoxycholate] mixed with 1% protease inhibitor and phosphatase inhibitor. The cell lysis was performed at 4 °C for 40 min, followed by centrifugation at 12,000 rpm/min for 25 min. The supernatants were harvested and subjected to protein concentration measurement using the BCA assay, followed by adding the loading buffer, mixing, and separating the proteins in the mixture on a 10% SDS-PAGE gel. The proteins separated on the gel were transferred to PVDF membranes, then the membrans had been blocked with 5% non-fat milk for 1 h at RT. Next, the membranes with proteins were incubated overnight with the primary antibody solution at 4 °C. After thorough washing followed by incubation with the HRP-conjugated secondary antibody solution for 1 h at RT, the protein bands were visualized and imaged using the ECL detection system (Bio-Rad Laboratories).

### Immunohistochemistry

Tissues that were formalin-fixed, paraffin-embedded, and excised into 4-μm slices were examined by immunohistochemistry (IHC). After microwave heat-based antigen retrieval, the tissue sections were incubated overnight with primary antibodies and then with the secondary antibody. Next, the tissue sections were allowed to react with diaminobenzidine to develop color. Hematoxylin was added to the slides as a counterstain. In a double-blind process, the expression levels of GPER1 and SCD1 in the samples on the slides were determined, and the scoring system was logically modified as previously reported (Huang et al. 2018).

### Tissue immunofluorescence

Polychrome immunofluorescence Kit (RC0086, Recordbio) was used according to the manufacturer’s instructions. After antigen retrieval, the tissue sections were sequentially incubated with primary antibodies overnight and then the secondary antibodies. Next, the fluorescence color development reaction was conducted. Each experiment was performed in triplicate. Finally, DAPI was added to stain the nuclei. Images were acquired using a scanner from Servicebio.

### CCK8 assay

Cells were seeded in the wells of 96-well plates (same cell density in each well). The medium containing the drug was refreshed the next day, and the cell culture was continued at 37 °C for the next 48 h. Afterward, the cells were incubated for 30 min in a fresh medium containing 10% CCK8 reagent. The absorbance in each well was measured at 450 nm with a microplate scanner.

### Plate cloning formation assay

A total of 1000 cells were seeded uniformly across the wells of 6-well plates, and the drug-containing medium was refreshed first on the third day and then every four days. The experiments were terminated when the appropriate cell mass was reached in 15th days. Then, the cells were fixed in 4% paraformaldehyde for ten minutes, followed by 15 min of staining using the crystal violet dye solution. After a thorough wash, images were acquired using a scanner.

### Cell viability/cytotoxicity assay

Cells were seeded in the wells of 12-well plates (same cell density in each well) and the medium containing the drug was added the next day, and the cell culture was continued at 37 °C for the next 48 h. After removing the medium, an appropriate volume of Calcein AM/PI assay solution (C2015S, Beyotime) was added to plate and the cell was incubated at 37℃ in the dark for 20 min. The fluorescent microscope was used for observing and recording the staining effect.

### Lipid peroxidation test

Lipid Peroxidation MDA Assay Kit (S0131M, Beyotime) was used according to the manufacturer’s instructions. The protein solutions were prepared using the lysate, and the BCA assay was performed. At 100 °C and in an acidic atmosphere, MDA and TBA combine to generate a red MDA-TBA adduct, and the resulting absorbance was measured at 532 nm using a microplate reader. The initial MDA content in the samples was expressed in protein content per weight.

BODIPY™ 581/591 C11 (D3861, Thermo Fisher) was used in the lipid peroxidation test. First, BODIPY™ 581/591 C11 was diluted to 10 μM. Next, 1 mL of the staining solution was added to each well of the 6-well plate, sufficient to cover the cells in the wells, following which the plate was placed in an incubator at 37 °C for 1 h. Subsequently, the cells were collected into flow tubes after washing away the excess staining solution using PBS. Afterward, the cells were resuspended with PBS containing 5% FBS. A flow cytometric analysis was performed, and the corresponding signals were measured at the FITC and PE channels.

### Tumor formation assay

A549 cells (density = 4 × 10^6^ cells) were infected with shGPER1 or negative control shRNA and then injected subcutaneously into 6-week-old nude mice. The growth status of these nude mice and the change in the size of the transplanted tumor were observed daily. In addition, the size change in the subcutaneous tumor of the nude mice was measured every 4 days beginning from the 4th day. Cisplatin treatment (2 mg/kg, i.p., q4d) was administered 5 times. The mice were euthanized at 28 days, and the tumors were retrieved for measurements after paraffin embedding and IHC staining.

### FerrDb database

FerrDb (www.zhounan.org/ferrdb) (Zhou and Bao, 2020) is the world’s first database dedicated to the study of the association between ferroptosis regulators and diseases. FerrDb comprises seven independent datasets. In the present study, the “suppressor” dataset of FerrDb was searched for genes that prevent ferroptosis, and the top 8 genes were selected for validation.

### Statistical analysis

The result data were analyzed using GraphPad Prism 9.03 statistical software. Unless otherwise stated, each experiment was conducted in triplicate, with comparable results. The unpaired two-tailed Student’s t-test and one-way analysis of variance were performed for inter-group comparisons. All results are expressed as mean ± SD (standard deviation). A *p*-value of < 0.05 was considered statistically significant. The relationship between GPER1 expression level and the prognosis of NSCLC patients was analyzed using the online database KMplotter (http://www.kmplot.com/lung) (Gyorffy et al. 2013).

## Results

### GPER1 promoted anti-oxidative stress and anti-lipid peroxidation in NSCLC cells

**Fig. 1:**
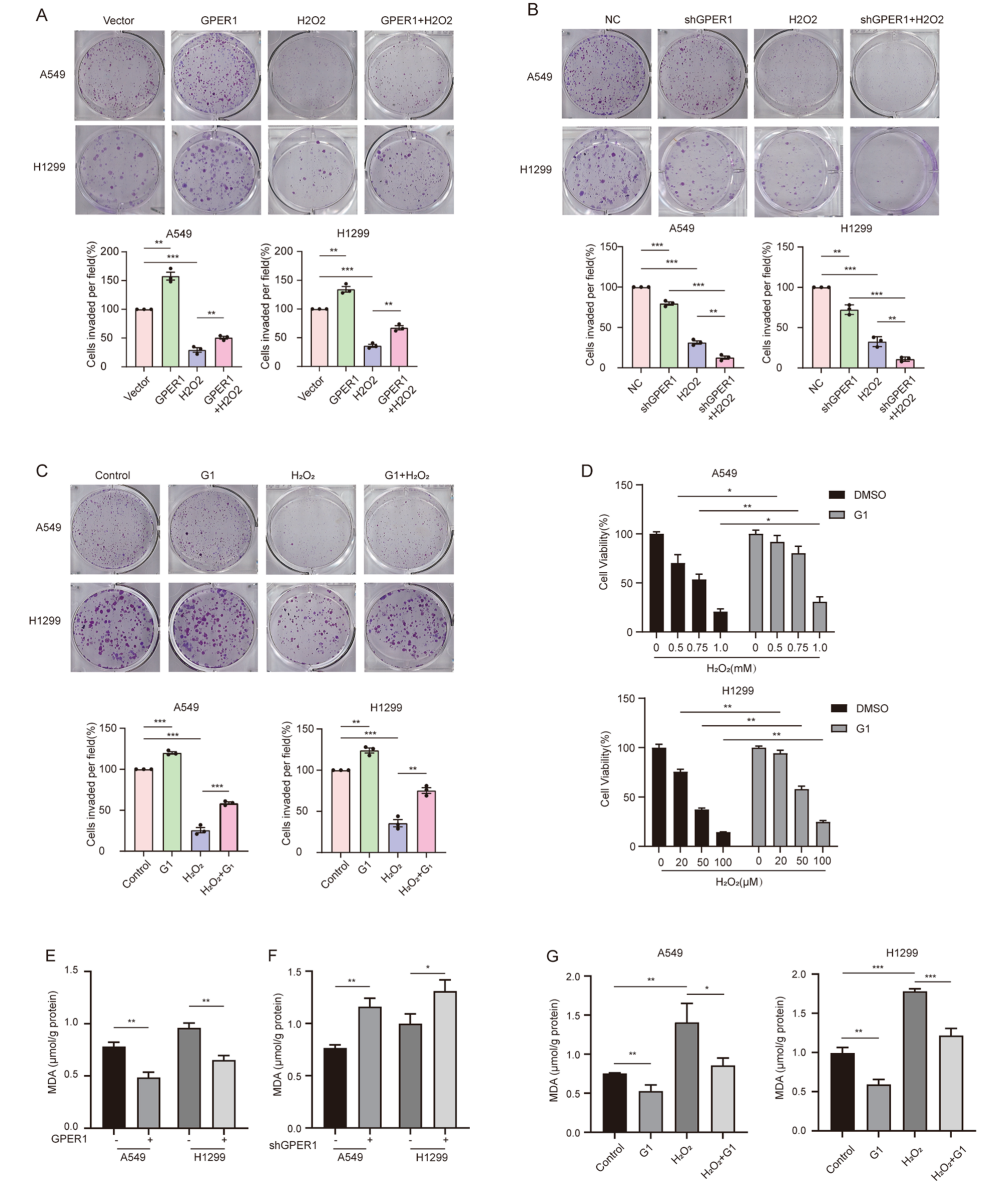
GPER1 promoted anti-oxidative stress and anti-lipid peroxidation in NSCLC cells. (**A**) The proliferation of A549 (250 μM) and H1299 (10 μM) cells with GPER1 overexpression and vectors that were treated with H_2_O_2_ was determined using the plate cloning formation assay. (**B**) The proliferation of GPER1-knockdown and negative control A549 (250 μM) and H1299 (10 μM) cells treated with H_2_O_2_ was determined using the plate cloning formation assay. (**C**) The proliferation of A549 and H1299 cells (as stated earlier) treated with G1 and H_2_O_2_ was determined using the plate cloning formation assay. (**D**) Cell viability was determined using the CCK8 assay after 48 h of G1 (1 μM) and H_2_O_2_ treatments in A549 and H1299 cells. (**E**, **F**) MDA levels in A549 and H1299 cells with GPER1 overexpression or knockdown. (**G**) MDA levels in A549 and H1299 cells after treatment with G1, H_2_O_2_, or both for 48 h. The results are presented as the mean ± SD. n = 3; *P < 0.05, **P < 0.01, ***P < 0.001

Anti-oxidative stress typically mediates tumor survival and proliferation. To explore the role of GPER1 in regulating oxidative stress, hydrogen peroxide (H_2_O_2_) was used to induce intracellular lethal oxidative stress in NSCLC cells, followed by the evaluation of whether GPER1 would exert an impact on cell viability. GPER1 overexpression improved the viability of A549 cells treated with 0.5, 0.75, and 1.0 mM H_2_O_2_ and H1299 cells treated with 20, 50, and 100 μM H_2_O_2_ (Figure S1A). In addition, GPER1 overexpression abrogated the reduction in cell proliferation in response to treatment with H_2_O_2_ (Fig. 1A). Conversely, GPER1 knockdown exacerbated the cytotoxicity of H_2_O_2_ and reduced the viability of the A549 and H1299 cell lines (Figure S1B). GPER1 knockdown significantly aggravated the damage to cell proliferation caused by H_2_O_2_ (Fig. 1B). Similar results were observed when the GPER1 agonist G1 was used (Fig. 1C and D). Moreover, GPER1 knockdown increased MDA levels, while GPER1 overexpression inhibited lipid peroxidation in the A549 and H1299 cell lines (Fig. 1E and F). G1 decreased the levels of MDA, a marker of lipid peroxidation. G1 also blocked the increase in the levels of MDA in response to treatment with H_2_O_2_ (Fig. 1G).

### GPER1 protected NSCLC cells from ferroptosis

**Fig. 2:**
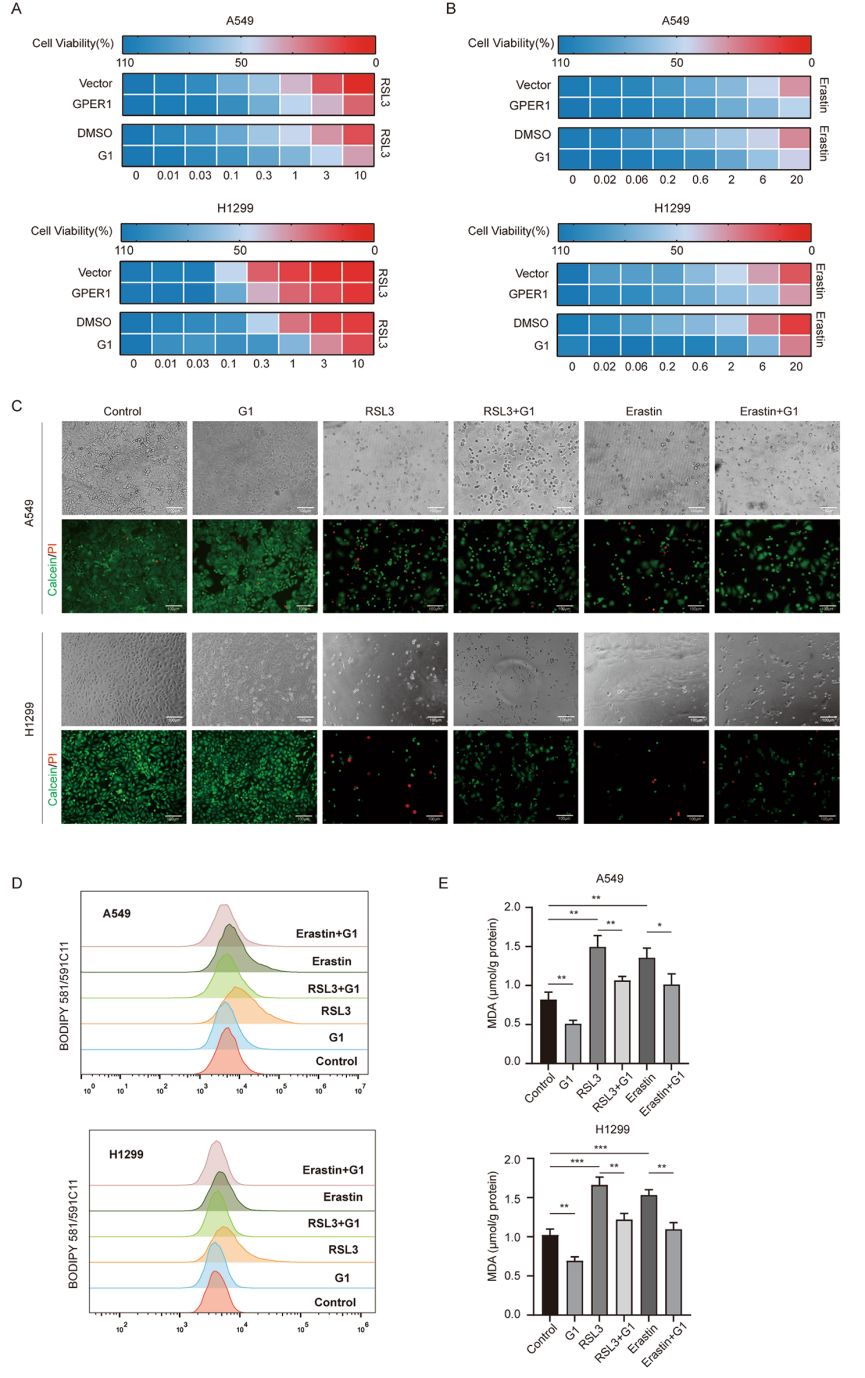
GPER1 protected NSCLC cells from ferroptosis. (**A**, **B**) Heatmap showing that GPER1 reduced sensitivity to the ferroptosis inducers RSL3 and Erastin. Darker blue indicates higher cell viability, and darker red indicates worse viability. The number at the bottom of the heatmap indicates the concentration of G1. (**C**) Representative phase-contrast images and viability staining (green indicates live cells, and red indicates dead cells) of A549 and H1299 cells treated with G1, RSL3, or Erastin. (**D**) The fluorescence of BODIPY 581/591C11 in A549 and H1299 cells treated with G1, RSL3, or Erastin, was determined using flow cytometry. (**E**) MDA levels in A549 and H1299 cells treated with G1, RSL3, or Erastin. The results are presented as the mean ± SD. n = 3; *P < 0.05, **P < 0.01, ***P < 0.001

Lipid peroxidation is the most significant indicator of ferroptosis, and GPER1 reportedly reduces lipid peroxidation. Therefore, it was hypothesized that GPER1 could inhibit ferroptosis. To confirm this hypothesis, the effect of GPER1 on the cytotoxicity of ferroptosis inducers (RSL3 and Erastin) was evaluated in the present study. GPER1 overexpression and G1 treatment inhibited the cytotoxicity of RSL3 and Erastin on the A549 and H1299 cell lines (Fig. 2A and B, Figure S1C), and RSL3 exhibited more robust cytotoxicity. G1 also protected the cells from ferroptosis (Fig. 2C). Moreover, changes in lipid peroxidation levels were examined under the same conditions, and the results demonstrated that G1 significantly attenuated RSL3- and Erastin-induced lipid peroxidation (Fig. 2D and E). These results demonstrated that GPER1 protected NSCLC cells from ferroptosis.

### GPER1 prevented ferroptosis by upregulating SCD1

**Fig. 3:**
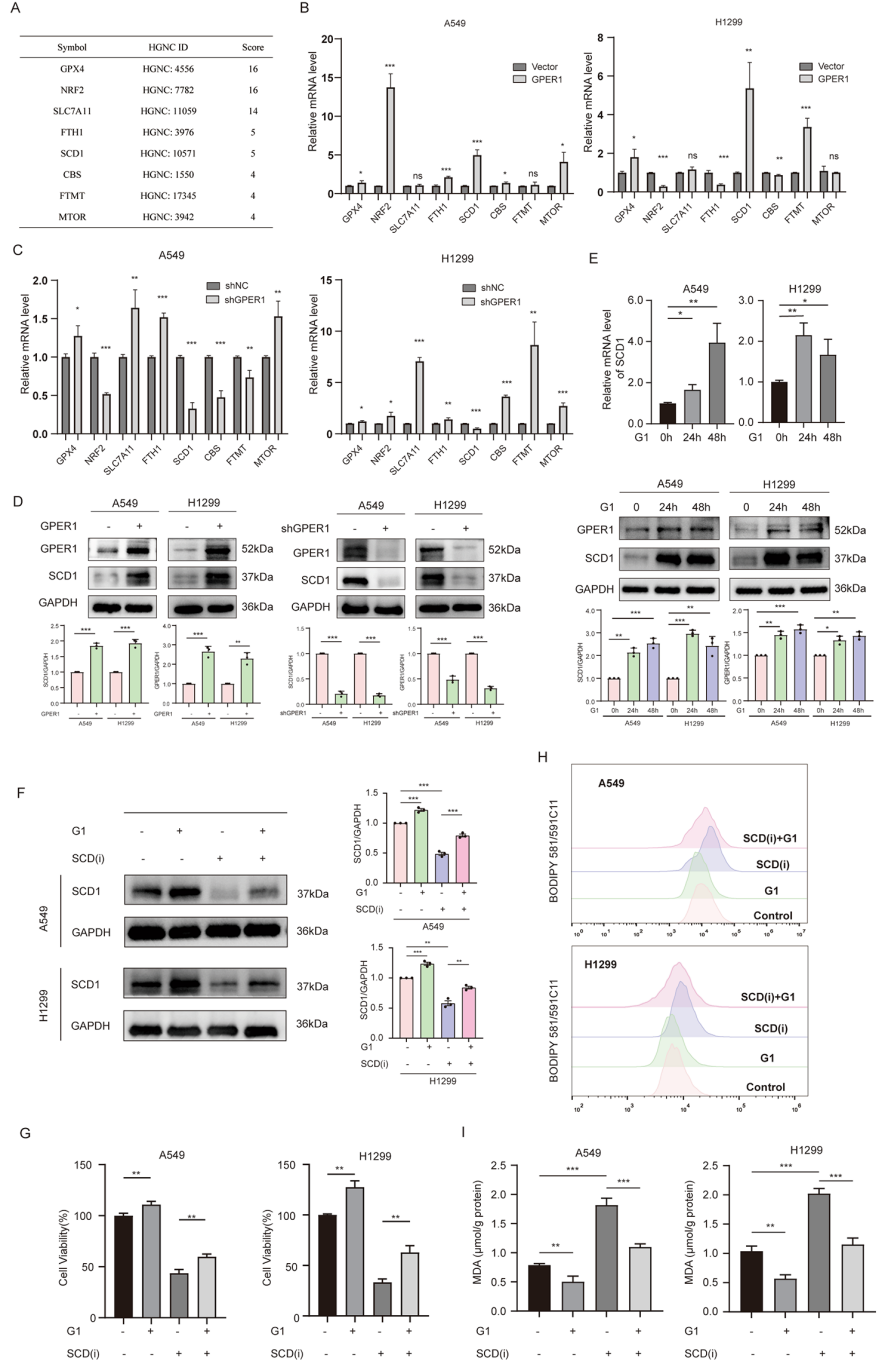
GPER1 prevented ferroptosis by upregulating SCD1. (**A**) The top 8 ferroptosis suppressors were searched in the FerrDb database. (**B**) The mRNA levels of the 8 ferroptosis suppressors after GPER1 overexpression in A549 and H1299 cells. (**C**) The mRNA levels of the 8 ferroptosis suppressors after GPER1 knockdown in A549 and H1299 cells. (**D**) The protein levels of SCD1 in GPER1-overexpressing or knockdown cells. (**E**) The mRNA and protein levels of SCD1 in A549 and H1299 cells treated with G1 for 24 and 48 h. (**F**) GPER1 and SCD1 levels were determined in A549 and H1299 cells that were treated with G1, A939572, or both for 48 h. (**G**) Cell viability was evaluated using the CCK8 assay. (**H**) The fluorescence of BODIPY 581/591C11 was determined using flow cytometry. (**I**) MDA levels in A549 and H1299 cells that were treated with G1, A939572, or both for 48 h. The results are presented as the mean ± SD. n = 3; *P < 0.05, **P < 0.01, ***P < 0.001

To determine how GPER1 inhibits ferroptosis, the FerrDb database was searched, and the top 8 genes in the ferroptosis suppressor gene module were selected for further analysis (Fig. 3A). The fold changes in mRNAs related to these top 8 genes in the A549 and H1299 cell lines in response to GPER1 overexpression and knockdown were assessed. When GPER1 was overexpressed, the mRNA expression levels of GPX4, NRF2, fth1, and SCD1 were significantly increased in A549 cells, while GPX4, SCD1, and FTMT expression was significantly increased in H1299 cells. In addition, when GPER1 was knocked down, only the expression of SCD1 was decreased in A549 and H1299 cells. These results suggest that GPER1 positively regulates SCD1 expression (Fig. 3B and C). SCD1 protein levels also exhibited the same trend (Fig. 3D). Moreover, G1 treatment for 24 and 48 h increased the mRNA and protein levels of SCD1 in A549 and H1299 cells (Fig. 3E). Therefore, it was hypothesized that GPER1 could prevent ferroptosis by promoting the expression of SCD1. Accordingly, the SCD1-specific inhibitor A939572 was used to block the SCD1 protein, followed by analysis. The results demonstrated that G1 could prevent the reduction in SCD1 protein expression caused by A939572 (Fig. 3F). G1 also promoted cell viability and inhibited the cytotoxicity of A939572 (Fig. 3G). Similarly, A939572 increased intracellular lipid peroxidation levels, while G1 inhibited lipid peroxidation caused by A939572 (Fig. 3H and I). Collectively, these results suggest that GPER1 upregulates SCD1 expression to prevent ferroptosis in NSCLC cells.

### GPER1 promoted SCD1 transcription via SREBP1

**Fig. 4:**
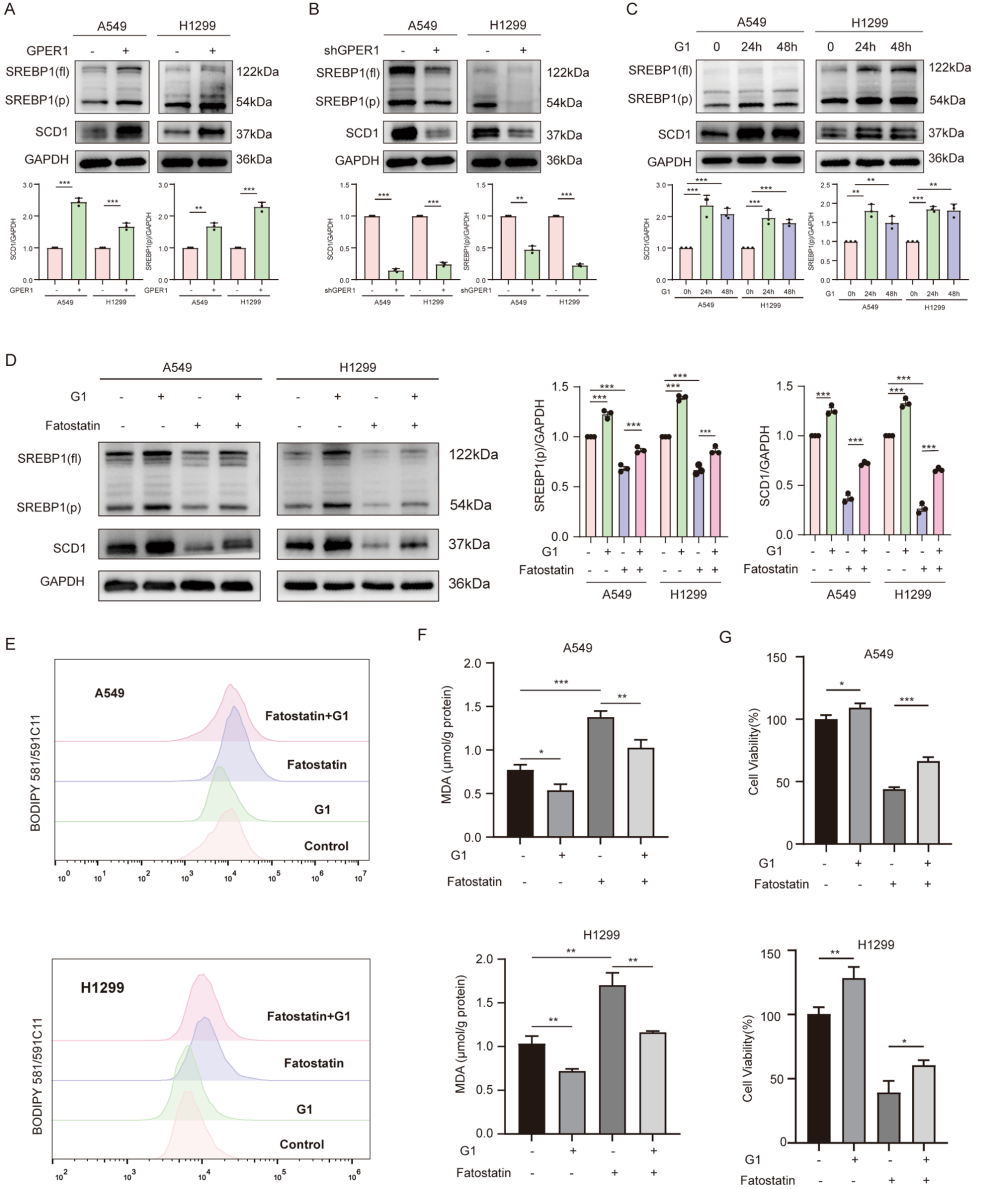
GPER1 promoted SCD1 transcription via SREBP1. (**A**, **B**) The protein levels of SREBP1(p) in GPER1-overexpressing or knockdown cells. (**C**) The protein levels of SREBP1 (p) in A549 and H1299 cells treated with G1 for 24 and 48 h. (**D**) SREBP1(**p**) levels were determined in A549 and H1299 cells that were treated with G1, fatostatin, or both for 48 h. (**E**) The fluorescence of BODIPY 581/591C11 in A549 and H1299 cells that were treated with G1, fatostatin, or both for 48 h was analyzed using flow cytometry. (**F**) MDA levels in A549 and H1299 cells that were treated with G1, fatostatin, or both for 48 h. (**G**) The viability of A549 and H1299 cells that were treated with G1, fatostatin, or both for 48 h was evaluated using a CCK-8 assay. The results are presented as the mean ± SD. n = 3; *P < 0.05, **P < 0.01, ***P < 0.001

As a membrane receptor, GPER1 is not directly involved in the transcription of any target genes. Therefore, it was hypothesized that GPER1 promotes SCD1 transcription through the activation of transcription factors that affect SCD1. Sterol regulatory element binding protein 1 (SREBP1) is a classic transcription factor that affects SCD1. Whether SREBP1 was involved in the regulation of SCD1 via GPER1 was investigated. The expression of full-length SREBP1(fl.) and its activated form SREBP1(p) were evaluated in the A549 and H1299 cell lines after GPER1 overexpression and knockdown. SREBP1(p) was significantly increased in GPER1-overexpressing cell lines and reduced in response to GPER1 knockdown (Fig. 4A and B). G1 treatment also increased the levels of SREBP1(p) in A549 and H1299 cells (Fig. 4C). Furthermore, fatostatin, an effective inhibitor of SREBP1 activation, was used to repress SREBP1(p) and inhibited the expression of SCD1. G1 significantly prevented the decrease in the levels of SREBP1(p), resulting in SCD1 induction by fatostatin (Fig. 4D). Fatostatin significantly increased intracellular lipid peroxidation levels, while G1 antagonized lipid peroxidation caused by fatostatin in A549 and H1299 cells (Fig. 4E and F), and the same held true for cell viability (Fig. 4G).

### GPER1 promoted SREBP1-SCD1 via PI3K/AKT/mTOR signaling

**Fig. 5:**
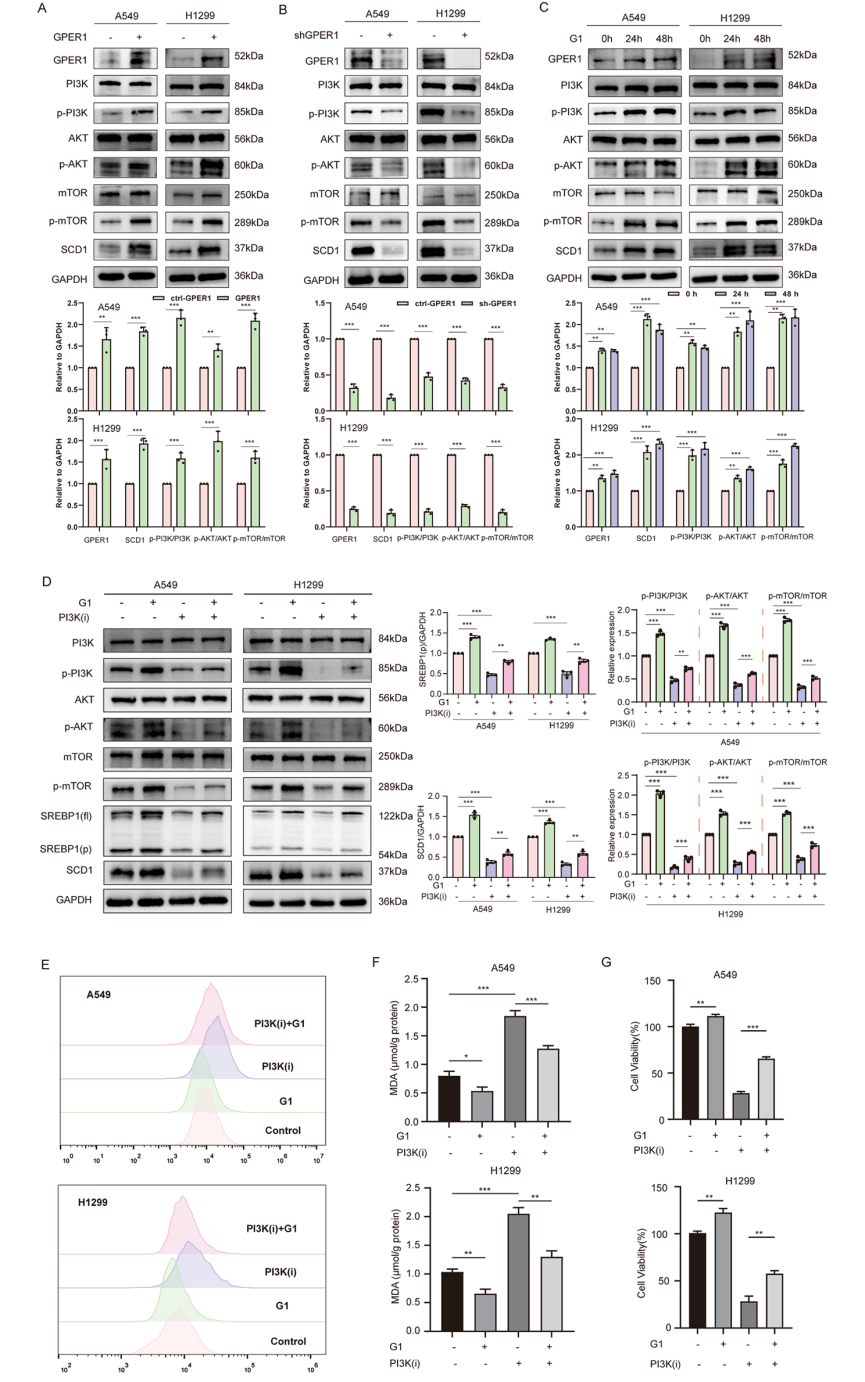
GPER1 promoted SREBP1-SCD1 via PI3K/AKT/mTOR signaling. (**A**, **B**) The activation of PI3K/AKT/mTOR signaling (the protein levels of p-PI3K, p-AKT, and p-mTOR) in the GPER1-overexpressing or knockdown cells. (**C**) The activation of PI3K/AKT/mTOR signaling (the protein levels of p-PI3K, p-AKT, and p-mTOR) in A549 and H1299 cells treated with G1 for 24 and 48 h. (**D**) The levels of SREBP1 (fl.), SREBP1 (p), and SCD1 and the activation of PI3K/AKT/mTOR signaling (the protein levels of p-PI3K, p-AKT, and p-mTOR) were determined in A549 and H1299 cells that were treated with G1, PI3K(i) (NSC781406), or both for 48 h. (**E**) The fluorescence of BODIPY 581/591C11 in A549 and H1299 cells that were treated with G1, PI3K(i) (NSC781406), or both for 48 h, as analyzed using flow cytometry. (**F**) MDA levels in the A549 and H1299 cells that were treated with G1, PI3K(i) (NSC781406), or both for 48 h. (**G**) The viability of A549 and H1299 cells that were treated with G1, PI3K(i) (NSC781406), or both for 48 h, determined using the CCK8 assay. The results are presented as the mean ± SD. n = 3; *P < 0.05, **P < 0.01, ***P < 0.001

GPER1 promotes tumor progression mainly by interacting with carcinogenic signaling pathways, such as the EGFR/ERK1/2 pathway, the PI3K/AKT/mTOR signaling pathway, and the MAPK/ERK signaling pathway. It was further revealed that excessive activation of the PI3K/AKT/mTOR signaling pathway could enhance ferroptosis resistance in lung cancer cells, and this was achieved by inducing the expression of SREBP1/SCD1. Therefore, it was hypothesized that GPER1 promoted SREBP1/SCD1 through the activation of PI3K/AKT/mTOR signaling. To verify this hypothesis, experiments were conducted, and GPER1 overexpression promoted activation of the PI3K/AKT/mTOR signaling pathway, while GPER1 knockdown reduced the expression of the PI3K/AKT/mTOR signaling pathway (Fig. 5A and B). G1 also significantly increased the expression of the PI3K/AKT/mTOR signaling pathway (Fig. 5C). NSC781406, a PI3K activity inhibitor, was used to block PI3K/AKT/mTOR signaling. The levels of SREBP1(p) and SCD1 were significantly reduced by NSC781406, and G1 prevented the decrease in the protein levels of SREBP1(p) and SCD1 caused by NSC781406 (Fig. 5D). NSC781406 significantly increased intracellular lipid peroxidation levels, while G1 antagonized lipid peroxidation caused by NSC781406 in A549 and H1299 cells (Fig. 5E and F); the same held true for cell viability (Fig. 5G). These results collectively indicated that GPER1 promoted SCD1 transcription by activating PI3K/AKT/mTOR signaling.

### GPER1 correlated with SCD1 in NSCLC tissues

**Fig. 6:**
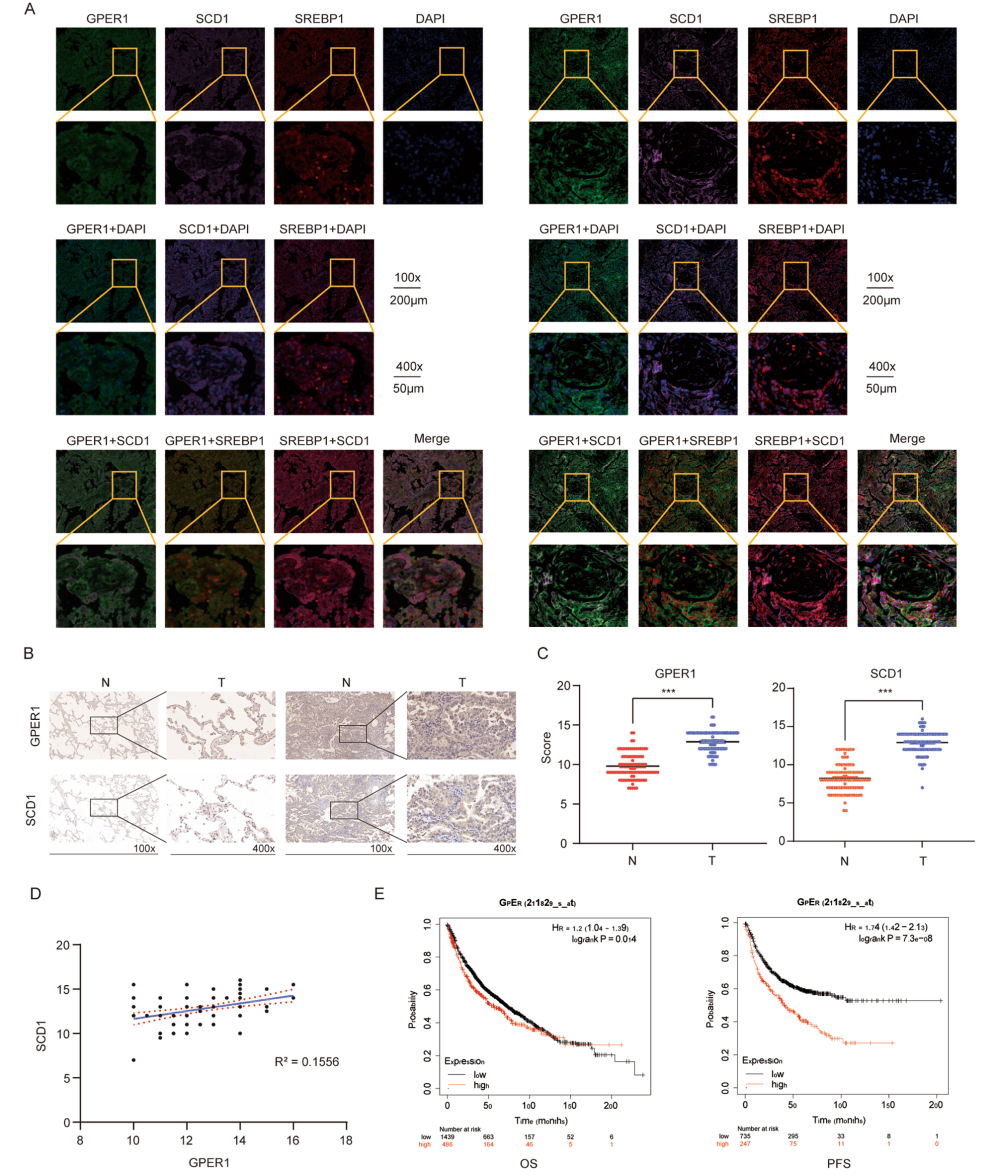
GPER1 correlated with SCD1 in NSCLC tissues. (**A**) An immunofluorescence assay was performed on GPER1 (Green, TYR-520), SCD1 (Purple, TYR-690), SREBP1 (Red, TYR-570) and nucleus (Blue, PADI) in the NSCLC tissue with different levels of GPER1 expression (the right panel shows higher levels and the left one shows lower levels). (**B**, **C**) The expression of GPER1 and SCD1 in tumor tissue and normal tissue were detected using immunohistochemistry. (**D**) The relationship between GPER1 and SCD1 expression in NSCLC tissue; R squared: 0.1556. (**E**) Overall survival (OS) and progression-free survival (PFS) of NSCLC cells with GPER1 expression. *P < 0.05, **P < 0.01, ***P < 0.001

Previous studies have demonstrated that GPER1 is upregulated in lung cancer tissues. In the present study, the relationship among GPER1, SCD1, and SREBP1 in NSCLC tissues was investigated. A tissue immunofluorescence assay was performed, and GPER1, SCD1, and SREBP1 were labeled with different fluorescence signals. The results indicated that when GPER1 levels were high, SCD1 and SREBP1 were abundantly expressed (Fig. 6A). The IHC staining results indicated that the expression levels of GPER1 and SCD1 in tumor tissues were significantly higher than those in corresponding normal tissues (Fig. 6B and C). Furthermore, the correlation between GPER1 and SCD1 expression was determined. Correlation analysis showed a positive correlation between GPER1 and SCD1 expression in NSCLC tissues (Fig. 6D). Next, the association between the clinicopathological characteristics of NSCLC and GPER1 expression was investigated. High GPER1 expression was substantially associated with poor overall survival (OS) and progression-free survival (PFS) in NSCLC, as revealed by the analysis of Kaplan‒Meier survival curves and the log-rank test (Fig. 6E).

### GPER1 deficiency potentiated the cytotoxic effect of cisplatin by inducing ferroptosis

**Fig. 7:**
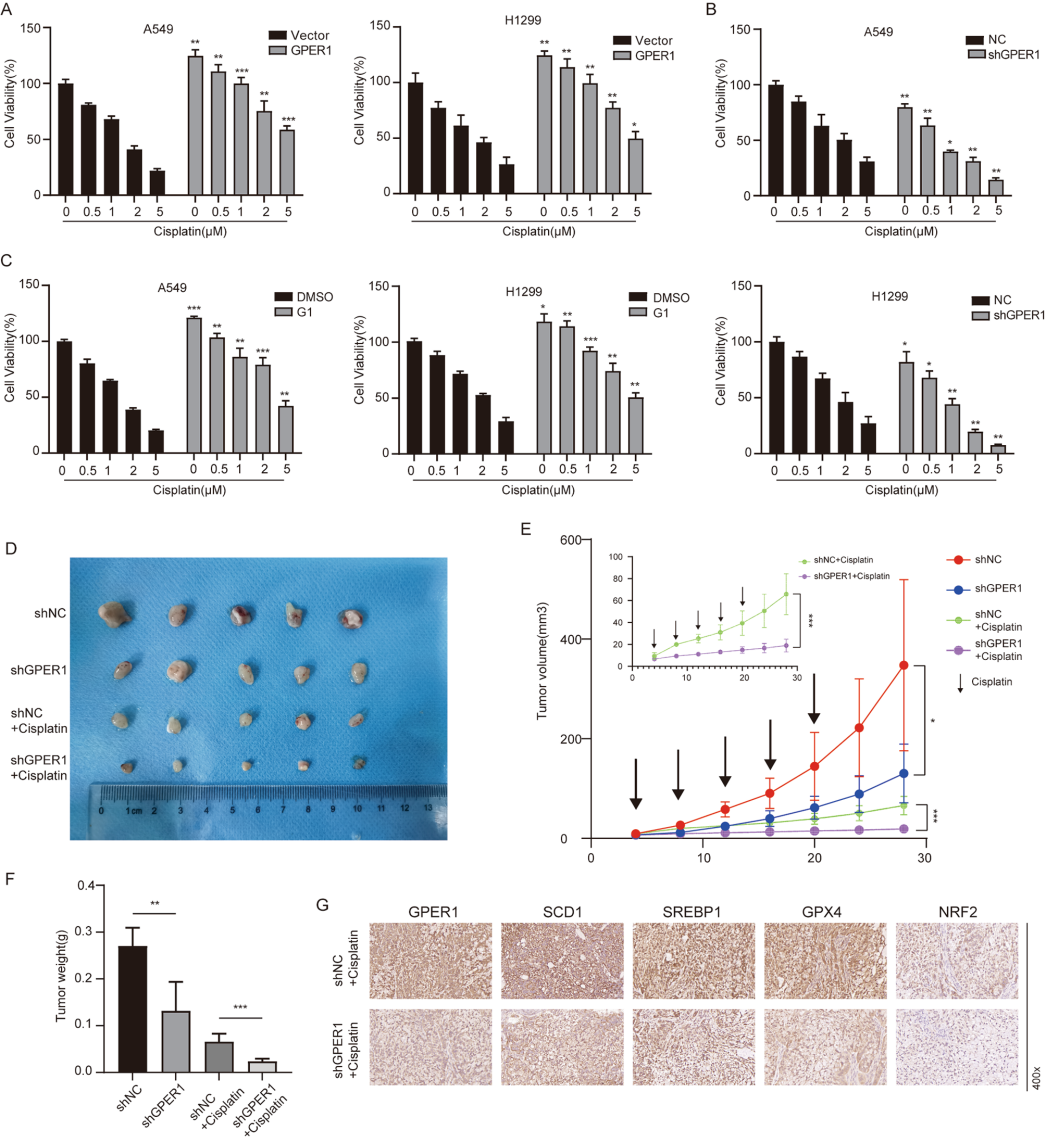
GPER1 deficiency potentiated the cytotoxic effect of cisplatin by inducing ferroptosis. (**A**, **B**) The viability of in A549 and H1299 cells with GPER1 overexpression or knockdown was determined using the CCK8 assay after treatment with cisplatin (0.5 μM, 1 μM, 2 μM, and 5 μM). (**C**) The viability of A549 and H1299 cells with or without G1 treatment was determined using the CCK8 assay after treatment with cisplatin (0.5 μM, 1 μM, 2 μM, and 5 μM). (**D**) Images of subcutaneous tumors from the indicated groups (n = 5 per group). (**E**) Tumor volumes in xenograft mice were measured at the indicated times. (**F**) Tumor weights in xenograft mice. (**G**) GPER1, SCD1, SREBP1, GPX4, and NRF2 expression in subcutaneous tumors from the indicated groups was determined using immunohistochemistry. The results are presented as the mean ± SD. n = 3; *P < 0.05, **P < 0.01, ***P < 0.001

Cisplatin is currently considered indispensable in the comprehensive treatment regimen for NSCLC. One of the cytotoxic mechanisms of cisplatin involves inducing intracellular oxidative stress. Studies have also demonstrated that cisplatin induces ferroptosis, and combination therapy with Erastin exerts a significant synergistic antitumor effect. Since GPER1 was shown to prevent ferroptosis in the present study, it was expected that the combination of GPER1 deficiency and cisplatin could enhance the antitumor activity of GPER1. To confirm this hypothesis, experiments were conducted, and GPER1 overexpression impaired the cytotoxic effects of cisplatin, while GPER1 knockdown enhanced the cytotoxic effects of cisplatin on A549 and H1299 cells (Fig. 7A and B). Combination treatment with G1 and cisplatin increased the viability of A549 and H1299 cells (Fig. 7C). Intriguingly, a subcutaneous graft model was established using A549 cells with GPER1 knockdown and negative control cells. GPER1 knockdown slowed tumor growth, resulting in reduced tumor size and weight (Fig. 7D, E and F). Moreover, GPER1 knockdown and cisplatin treatment exerted significant antitumor effects, and the tumor was in a state of growth arrest (Fig. 7D, E and F). Next, IHC staining of the subcutaneous graft was performed, and the results revealed that the expression levels of GPER1, SCD1, and SREBP1 were low in the GPER1 knockdown plus cisplatin treatment group. Correspondingly, GPX4 and NRF2, which are markers of ferroptosis, were also low (Fig. 7G). These results suggested that GPER1 deficiency enhanced the antitumor activity of cisplatin by promoting ferroptosis.

## Discussion

Substantial evidence indicates that estrogen is strongly linked to NSCLC. Specifically, estrogen triggers protumor effects by activating ERs. Initially, ERβ was believed to be the primary subtype in NSCLC, although antiestrogen therapy led to limited clinical efficacy due to the inhibition of ERβ signaling (Garon et al. 2018; Stabile et al. 2011). Consequently, GPER1, which is the most recently identified ER, has emerged as a target of therapeutic interest. In the present study, GPER1 was shown antagonize oxidative stress and reactive oxygen species. Additionally, it was demonstrated that inhibiting lipid peroxidation could maintain cell viability. Specifically, these findings suggested that GPER1 protected against iron death in NSCLC. Previous studies have shown that the GPER1 agonist G1 reduces ROS production in human umbilical vein endothelial cells stimulated by H_2_O_2_ and increases cell survival, while the GPER1 antagonist G15 further increases ROS production (Kong et al. 2014). Kaempferol increases the protein expression of GPER1, which, in turn, ameliorates atherosclerosis by decreasing intracellular ROS generation via the PI3K/AKT/Nrf2 pathway (Feng et al. 2021). In breast cancer, estrogen has been reported to inhibit ROS generation by inducing the expression of antioxidant genes (Cook et al. 2014; Tian et al. 2016). These findings and the hypothesis proposed in the present study were unexpected and were confirmed in NSCLC. Overall, the present study demonstrated that GPER1 in NSCLC promoted cell viability by inhibiting oxidation.

Ferroptosis may be regulated by a variety of factors and regulates cancer progression. A study investigated the role of RBMS1 in regulating ferroptosis in lung cancer through translational control of SLC7A11 (Zhang et al., 2021). However, few studies have examined estrogen and ERs in the regulation of ferroptosis. A study reported that ERα could prevent ferroptosis in breast cancer cells that had been exposed to ionizing radiation, and this was achieved through the NEDD4L/CD71 pathway (Liu et al. 2022). The present study, to the best of the author’s knowledge, is the first to report that GPER1 protects against ferroptosis in NSCLC.

SCD1 plays a crucial role in regulating iron death and can advance tumor growth by inhibiting ferroptosis. Recent studies have identified concurrent mutations in STK11 and KEAP1 that appear to protect against ferroptosis and promote SCD1 dependence in lung cancer (Wohlhieter et al. 2020). Tumor resistance to ferroptosis, which is driven by SCD1 in cancer cells or by fatty acid binding protein-4 (FABP4) in the tumor microenvironment, promotes tumor recurrence (Luis et al. 2021). The oncogenic activation of PI3K/AKT/mTOR signaling suppresses ferroptosis via SREBP1/SCD1-mediated lipogenesis (Yi et al. 2020). It has been reported that 17β-estradiol induces SCD1 expression in estrogen receptor-positive breast cancer cells, which suggests an interaction between ERs and SCD1 (Belkaid et al. 2015). Since SCD1 is downstream of PI3K/AKT/mTOR signaling, GPER1 must be involved in activation of the MAPK, PI3K/AKT, and Notch1 signaling pathways and promote NSCLC progression, indicating that GPER1 has a regulatory effect on SCD1 expression (Liu et al. 2015; Shen et al. 2021; Yi et al. 2020). In the present study, it was demonstrated that GPER1 was required to prevent ferroptosis in NSCLC, and it increased SREBP1 expression rather than stimulated its processing through PI3K/ATK/mTOR signaling, thereby promoting SCD1 transcription.

Cisplatin is indispensable in tumor therapy. It has been reported that GPER1-mediated epithelial-mesenchymal transition induces cisplatin resistance in gastric cancer cells (Wang et al. 2020). Moreover, cisplatin can induce ferroptosis in A549 cells, and combination therapy with cisplatin and erastin exerts a significant synergistic antitumor effect (Guo et al. 2018). Our study also demonstrated that GPER1 could reduce cisplatin cytotoxicity and that treatments targeting GPER1 combined with cisplatin could effectively enhance the antitumor activity of cisplatin by enhancing ferroptosis. In conclusion, the present study provides novel insights into the role of GPER1 in NSCLC and a novel strategy for the treatment of NSCLC.

## Conclusion

The present study aimed to enhance our comprehension of estrogen signaling in the progression of NSCLC. Specifically, the findings suggest that GPER1 may be a promising target or biomarker for the treatment of NSCLC. We propose that GPER1 can prevent ferroptosis in NSCLC, which is achieved by promoting SCD1 transcription via PI3K/AKT/mTOR signaling. Furthermore, combining treatments that inhibit GPER1 with cisplatin is expected to enhance cisplatin’s antitumor activity by promoting ferroptosis in NSCLC. The present study provides a novel approach for treating NSCLC by targeting GPER1.

### Electronic Supplementary Material

Below is the link to the electronic supplementary material.

**Supplementary Material 1:** (**A**) The viability of in A549 and H1299 cells overexpressing GPER1 and the vector was determined using the CCK8 assay after 48 h of treatment with H2O2. (**B**) The viability of GPER1 knockdown and negative control A549 and H1299 cells was determined using the CCK8 assay after 48 h of treatment with H2O2. (**C**) The dose-viability curve revealed that GPER1 reduced sensitivity to the ferroptosis inducers RSL3 and Erastin. (**D**) GPER1, p-PI3K, p-AKT and p-mTOR expression in subcutaneous tumors in the GPER1-knockdown and control groups were determined using immunohistochemistry. The results are presented as the mean ± SD. n = 3; *P < 0.05, **P < 0.01, ***P < 0.001

## Data Availability

The datasets and data used in this study can be obtained from the official website or corresponding author.

## List of abbreviations

ACSL4 Acyl: CoA synthetase long-chain family member 4
DMSO: Dimethyl Sulfoxide
ER: Estrogen recptor
FABP4: Fatty acid binding protein-4
GPER1: G-protein-coupled estrogen receptor 1
GPX4: Glutathione peroxidase 4
H2O2: Hydrogen peroxide
HRP: Horseradish Peroxidase
IHC: Immunohistochemistry
NRF2: Nuclear factor-erythroid-2-related factor 2
NSCLC: Non-small cell lung cancer
PBS: Phosphate Buffer Saline
RNA: Ribonucleic Acid
ROS: Reactive oxygen species
SCD1: Stearoyl CoA desaturase 1
SREBP1: Sterol-regulatory-element binding protein 1
WB: Western Blotting
