# Supplementary figures and images for: G protein-coupled estrogen receptor activates PI3K/AKT/mTOR signaling to suppress ferroptosis via SREBP1/SCD1-mediated lipogenesis
Source: Mol Med. 2024 Feb 21;30:28. doi: 10.1186/s10020-023-00763-x (PMC10880371; doi:10.1186/s10020-023-00763-x)

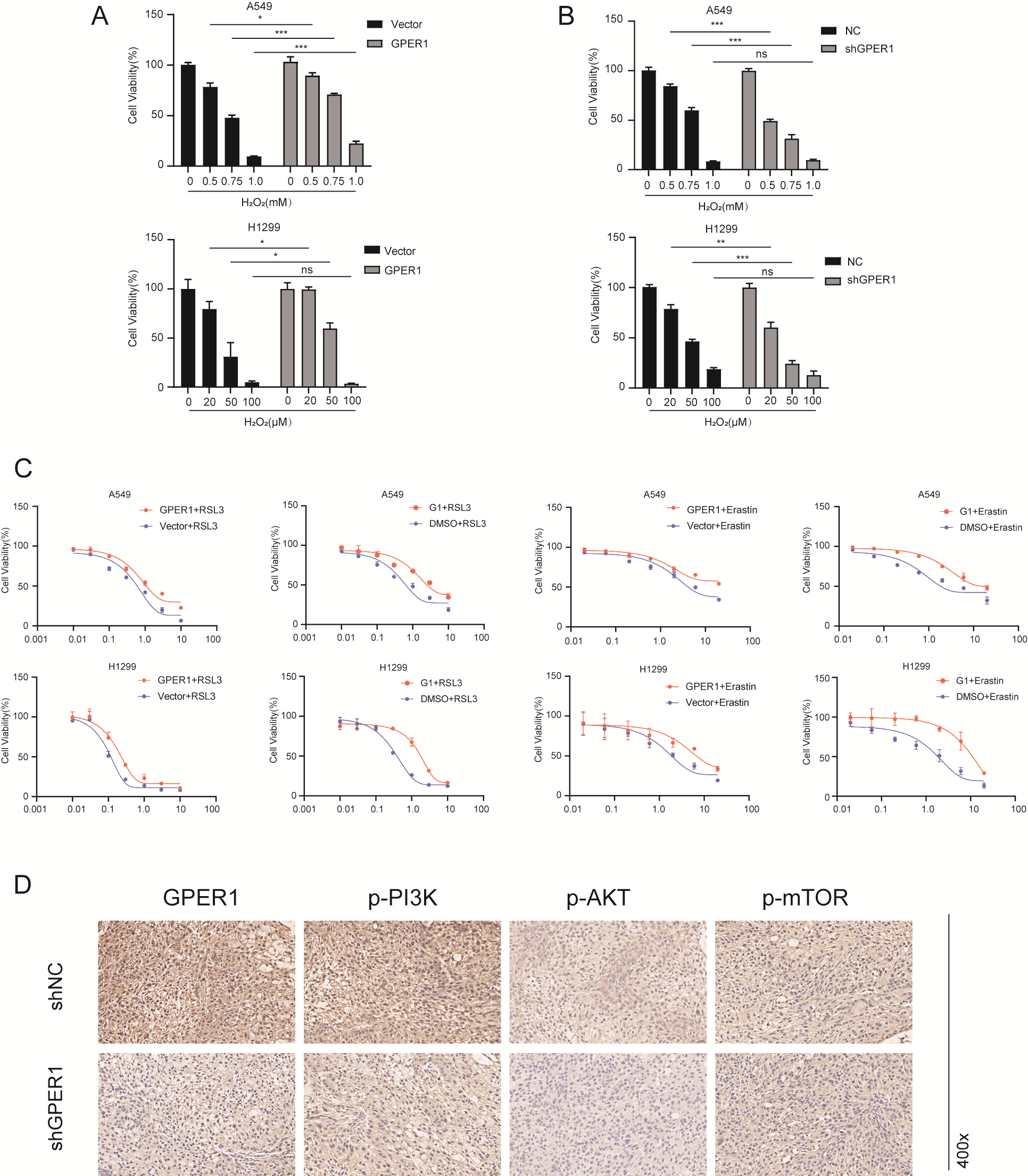

Supplement: Supplementary file 1 — Supplementary Material 1: (A) The viability of in A549 and H1299 cells overexpressing GPER1 and the vector was determined using the CCK8 assay after 48 h of treatment with H2O2. (B) The viability of GPER1 knockdown and negative control A549 and H1299 cells was determined using the CCK8 assay after 48 h of treatment with H2O2. (C) The dose-viability curve revealed that GPER1 reduced sensitivity to the ferroptosis inducers RSL3 and Erastin. (D) GPER1, p-PI3K, p-AKT and p-mTOR expression in subcutaneous tumors in the GPER1-knockdown and control groups were determined using immunohistochemistry. The results are presented as the mean ± SD. n = 3; *P < 0.05, **P < 0.01, ***P < 0.001 [file 10020_2023_763_MOESM1_ESM.png]
